# Dietary and lifestyle indices for hyperinsulinemia and colorectal cancer risk: a case-control study

**DOI:** 10.1186/s12876-023-03073-y

**Published:** 2023-12-11

**Authors:** Pegah Hadi Sicahni, Maede Makhtoomi, Kimia Leilami, Zainab Shateri, Farzaneh Mohammadi, Mehran Nouri, Niloofar Omidbeigi, Sanaz Mehrabani, Bahram Rashidkhani

**Affiliations:** 1 Department of Community Nutrition, School of Nutrition and Food Sciences, Nutrition and Food Security Research Center, Student Research Committee, Isfahan University of Medical Sciences, Isfahan, Iran; 2 Health Policy Research Center, Institute of Health, Shiraz University of Medical Sciences, Shiraz, Iran; 3 Student Research Committee, Shiraz University of Medical Sciences, Shiraz, Iran; 4 Department of Clinical Nutrition, School of Nutrition and Food Sciences, Shiraz University of Medical Sciences, Shiraz, Iran; 5 Department of Nutrition and Biochemistry, School of Medicine, Ilam University of Medical Sciences, Ilam, Iran; 6 Health Sciences Research Center, Mazandaran University of Medical Sciences, Sari, Iran; 7 Department of Clinical Nutrition, School of Nutrition and Food Sciences, Food Security Research Center, Isfahan University of Medical Sciences, Isfahan, Iran; 8 Department of Community Nutrition, Faculty of Nutrition and Food Technology, National Nutrition and Food Technology Research Institute, Shahid Beheshti University of Medical Sciences, Tehran, Iran

**Keywords:** Diet, Pattern, Lifestyle, Hyperinsulinemia, Colorectal cancer, Colorectal neoplasms, Iranian

## Abstract

**Background:**

The incidence of colorectal cancer (CRC) has increased in Iran, and determining the dietary patterns that can contribute to reducing or increasing the risk of CRC will help better control this disease. Therefore, in the current study, we assessed the association between the empirical lifestyle index for hyperinsulinemia (ELIH) and the empirical dietary index for hyperinsulinemia (EDIH) with the CRC odds.

**Methods:**

The present case (n = 71)-control (n = 142) study was carried out in several CRC surgical units of hospitals in Tehran, Iran. A semi-quantitative food frequency questionnaire containing 168 items was used to assess participants’ dietary intakes. The EDIH and ELIH scores were calculated by food groups and some variables such as body mass index and physical activity. Logistic regression models were applied to evaluate the association between the EDIH and ELIH scores with CRC odds.

**Results:**

According to baseline features of the study participants, there were significant differences between the controls and cases in ELIH score, fiber intake, taking aspirin, and family history of CRC in first- and second-degree relatives. Also, we found that the odds of CRC increased significantly in the last tertile compared to the first tertile in EDIH and ELIH in the adjusted model (odds ratio (OR) = 3.12; 95% confidence interval (CI): 1.30–7.48 and OR = 4.72; 95% CI: 1.15–19.39, respectively).

**Conclusions:**

In conclusion, the result of this study indicated that CRC odds was significantly greater in subjects with higher EDIH and ELIH scores. Also, according to the results of this study, lifestyle and diet with insulinemic potential can influence the CRC risk.

**Supplementary Information:**

The online version contains supplementary material available at 10.1186/s12876-023-03073-y.

## Introduction

Colorectal cancer (CRC) ranks second among cancer-related deaths and is one of the most common cancers worldwide [1]. CRC accounted for 10% of cancer incidence worldwide in 2020 [2]. CRC is a multifactorial disease with some risk factors, including a history of disease in first-degree family members, obesity, alcohol consumption, and hyperlipidemia [3]. Additionally, insulin resistance and hyperinsulinemia seem to be involved in the etiology of CRC through the mitogenic action of insulin, which stimulates cell proliferation and inhibits apoptosis [4, 5].

Hyperinsulinemia and insulin resistance are thought to be significant underlying mechanisms that connect lifestyle choices and poor diet to the onset of numerous chronic conditions [6, 7]. Although some dietary factors affect hyperinsulinemia and insulin resistance, dietary patterns or indices considering the intricate interactions between food and nutrients may be better than single foods for investigating the diet-disease relationship [8, 9]. The glycemic index (GI) and insulin index (II) are the most common dietary indices that assess postprandial blood glucose levels and the response of insulin to foods, respectively; however, they cannot accurately reflect the impacts of diet on the response of insulin over time [10–12].

One food-based dietary index is the empirical dietary index for hyperinsulinemia (EDIH), which is based on food groups in the diet that are positively and negatively associated with hyperinsulinemia [12]. Body mass index (BMI) and physical activity are considered in the empirical lifestyle index for hyperinsulinemia (ELIH) alongside dietary intake [12].

A few studies previously evaluated the relationship between ELIH and EDIH with CRC risk. A study conducted using the Health Professionals Follow-Up Study (HPFS) and the Nurses’ Health Study (NHS) data demonstrated that CRC risk increased by 26% in higher EDIH scores [13]. The other study among women revealed that CRC risk was positively associated with EDIH and ELIH scores [14]. Another study showed that compared to patients with the lowest EDIH score, patients with the highest EDIH had poor survival from CRC [15]. These studies have shown that EDIH and ELIH can affect CRC risk.

The incidence of CRC has increased in Iran, and determining the dietary patterns that can contribute to reducing or increasing the risk of CRC will help better control this disease and reduce the incidence of CRC. Therefore, in the current study, we assessed the association between the ELIH and the EDIH with the CRC odds that has not been evaluated in Iranian people.

## Methods

### Study design

The present case-control study was carried out in several CRC surgical units of Ayatollah Taleghani, Imam Khomeini, Imam Hussein, and Shariati hospitals in Tehran, Iran, between September 2008 and January 2010. It was a case-control study that involved several surgical units specializing in CRC. The case group was people diagnosed with CRC (maximum six months before the interview), and their diagnosis was confirmed by sigmoidoscopy and endoscopy biopsy. Patients were between the ages of 40 and 75 and had no history of malignancy in other parts of the body, inflammatory bowel disease, or familial adenomatous polyposis. Also, we excluded individuals with other types of cancer besides CRC and those who underwent emergency resection for CRC and whose diagnosis was verified after the surgery.

The control group participants were simultaneously selected from the same hospitals as the case group. The control group consisted of patients with acute, non-cancerous illnesses unrelated to their diet. The control group included individuals with osteoarticular disorders, sprains and fractures, skin diseases, nose or eye disorders, acute surgical conditions, disk disorders, injuries, and trauma. Two people from the control group were matched with one patient from the case group regarding gender and age (within five-year categories). Two were randomly selected if there were more than two potential control group participants. All subjects were required to provide written informed consent before the interview. Details of the current study have been previously published [16, 17].

**Fig. 1:**
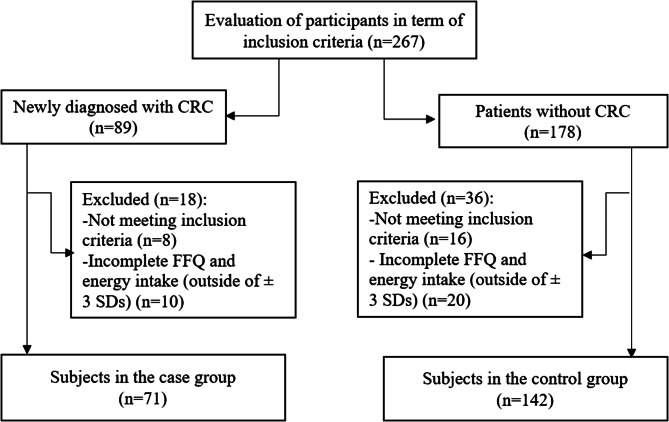
Flowchart of the study

In total, many patients (178 controls and 89 cases) were examined for the study, and 16 from the control group and eight from the case group were excluded due to the ineligibility of the inclusion criteria. Additionally, 20 controls and 10 cases were excluded because of unfinished food frequency questionnaire (FFQ) and total energy intake (outside of ± 3 standard deviations (SDs) from the mean). As a result, 142 people in the control group and 71 in the case group were examined (Fig. 1). The Ethics Committee of Shiraz University of Medical Sciences approved the current study (IR.SUMS.SCHEANUT.REC.1401.011).

### Dietary intake

The current study used a semi-quantitative FFQ containing 168 items to assess participants’ dietary intakes (Supplementary File 1). The questionnaire was completed by trained dietitians, and previous research has demonstrated that it has high reproducibility and validity in the Iranian population [18]. Participants in the control group completed the questionnaire based on their food intake one year before the interview. In comparison, CRC patients completed the questionnaire based on their food intake one year before their cancer diagnosis. Healthcare professionals assisted patients in estimating their food intake with a set of home measuring devices, including tablespoons, bowls, plates, cups, spatulas, teaspoons, and glasses for the portion size of each food item. Then, all data were turned into grams, and the consumption of each food was calculated by multiplying the frequency of daily consumption by the portion size. The energy content of the foods was specified through the database of Nutrient Composition of Iranian Foods. The researchers used the United States Department of Agriculture (USDA) food composition data for foods with no available data.

The EDIH score is determined by considering two categories of food components: negative and positive determinants. Negative determinants include leafy and green vegetables, coffee, high-fat dairy, and whole fruits. On the other hand, positive determinants comprise margarine, poultry, processed meat, red meat, French fries, eggs, tomatoes, high-energy beverages, butter, and low-fat dairy. Each of these groups is assigned a specific weight, which was previously determined in a study by Tabung et al. [12]. These weights are then multiplied by the corresponding food components, and the scores are summed up and then divided by 1000 to decrease the scores’ magnitude, making it easier to describe the results.

The ELIH score is almost more general and is calculated by considering a group of direct determinants, such as red meat, butter, margarine, fruit juice, and BMI, as well as inverse determinants such as physical activity, salad dressing, snacks, high-fat dairy products, whole fruits, and coffee. The ELIH score is computed similarly to the EDIH score, as mentioned [19]. The weight of each components of two indices was reported in Supplementary File 2.

### Covariates

A checklist was used to collect data including participants’ socioeconomic status, smoking habits, medication use, cooking methods, and family history of CRC (Supplementary File 3). A trained interviewer completed the checklist. It is worth noting that alcohol use is illegal in Iran, and participants were not asked about their alcohol intake in this study, as many patients declined to answer questions on this topic. The physical activity level of the participants was also extracted by a trained interviewer. The activity of two groups for the previous year was assessed by the International Physical Activity Questionnaire (IPAQ). This questionnaire contains the level of physical activity of the participants based on the metabolic equivalent of task (MET)-hours/day [20].

Standard methods were used to measure height and weight with a precision of 0.1 cm and 0.1 kg, respectively, without shoes and wearing minimal clothes [21]. For people hospitalized for a long time or who underwent surgery, their admission weight was taken as the current weight to computed BMI. For bedridden individuals, the recumbent length was measured. BMI was specified by dividing weight (kg) by height (m) ^2^. By a validated self-reported physical activity questionnaire, the physical activity level was assessed [22]. It was based on activity during the last year for controls and activity in the year before diagnosis for cases. Each individual’s MET was calculated as the time spent on different activities (MET-hours/day).

### Statistical analysis

The SPSS software (version 26.0) was applied to statistical analyses. The Kolmogorov-Smirnov test was used to measure the normality of variables. The basic characteristics of the control group and CRC patients were compared using the chi-square test for categorical variables and independent samples T-test or Mann-Whitney for continuous variables. Logistic regression models were applied to assess the association between EDIH and ELIH scores with CRC odds. Two crude (M1) and adjusted (M2) models were used to associate two EDIH and ELIH indices with CRC odds. In the adjusted model, the effect of confounding variables such as smoking, BMI, physical activity, energy intake, family history of CRC in first- and second-degree relatives, and taking aspirin and ibuprofen were adjusted. Odds ratios (ORs) and 95% confidence intervals (CIs) were calculated, and a significance level of less than 0.05 was used.

## Results

In Table 1, the baseline features of the study participants are presented. There were significant differences between the case and control groups in taking aspirin, and family history of CRC in the first-and second-degree relatives (*P* < 0.05 for all).

**Table 1:** The baseline features of the study population

Variables	Cases (n = 71)	Controls (n = 142)	*P*-value
Age (year)	58.2 ± 10.4	57.7 ± 10.4	0.746
Physical activity (MET-h/day)	36.8 ± 3.6	36.7 ± 4.8	0.932
Income (dollar)	393.0 (253.0)	402.0 (302.0)	0.206
BMI (kg/m^2^)	27.6 ± 4.2	26.6 ± 4.2	0.362
Gender			0.558
Male	35 (49.3)	70 (49.3)	
Female	36 (50.7)	72 (50.7)	
Education			0.147
No formal education	28 (39.3)	36 (25.4)	
Elementary	22 (31.0)	45 (31.6)	
Junior/Senior high school	7 (9.9)	19 (13.4)	
Diploma/College/University	14 (19.7)	42 (29.6)	
Smoking			0.164
Never	57 (80.2)	101 (70.1)	
Former	8 (11.3)	15 (10.6)	
Current	6 (8.5)	26 (18.3)	
Family history of CRC in the first degree			**0.017**
Yes	7 (9.9)	3 (2.1)	
No	64 (90.1)	139 (97.9)	
Family history of CRC in the second degree			**0.006**
Yes	6 (8.5)	1 (0.7)	
No	65 (91.5)	141 (99.3)	
Ibuprofen			0.059
Yes	5 (7.0)	22 (15.5)	
No	66 (93.0)	120 (84.5)	
Aspirin			**0.016**
Yes No	1 (1.4) 70 (98.6)	14 (9.9) 128 (90.1)	

MET: metabolic equivalent of task, BMI: body mass index, EDIH: empirical dietary index for hyperinsulinemia, ELIH: empirical lifestyle index for hyperinsulinemia

Using independent samples T-test or Mann-Whitney for continuous and chi-square test for categorical variables

According to Table 2, the ELIH score, fiber intake, intake of butter, tomatoes, whole fruits, and high-energy beverages significantly differed between the control and case groups (*P* < 0.05 for all). But, the EDIH score, energy intake, red and processed meats, poultry, fishes, eggs, margarine, salad dressing, low- and high-fat dairy products, green leafy vegetables, fruit juices, coffee, and snacks intake were not significant between the two groups (P˃0.05 for all).

**Table 2:** The intake of food groups in the study population

Variables	Cases (n = 71)	Controls (n = 142)	*P*-value
	Median (IQR)	Median (IQR)	
EDIH score	0.5 ± 0.2	0.4 ± 0.2	0.122
ELIH score	1.3 ± 0.2	1.2 ± 0.2	**0.022**
Energy (kcal/day)	2262.3 ± 450.1	2255.2 ± 341.2	0.908
Fiber (g/day)	18.9 ± 2.3	20.4 ± 3.1	**<0.001**
Red meats (serving/day)	0.38 (0.29)	0.34 (0.33)	0.081
Processed meats (serving/day)	0.08 (0.18)	0.06 (0.18)	0.505
Poultry (serving/day)	0.29 (0.25)	0.28 (0.25)	0.630
Other fishes (serving/day)	0.16 (0.11)	0.16 (0.19)	0.579
Eggs (serving/day)	0.27 (0.18)	0.32 (0.18)	0.962
Margarine (serving/day)	0.00 (0.00)	0.00 (0.00)	0.574
Butter (serving/day)	0.21 (0.63)	0.05 (0.25)	**0.016**
Salad dressing (serving/day)	0.11 (0.23)	0.13 (0.21)	0.990
Low-fat dairy products (serving/day)	0.86 (1.03)	1.02 (1.08)	0.040
High-fat dairy products (serving/day)	0.50 (0.63)	0.55 (0.58)	0.300
Green leafy vegetables (serving/day)	0.36 (0.32)	0.43 (0.33)	0.137
Tomatoes (serving/day)	0.80 (0.47)	0.80 (0.47)	**0.009**
Whole fruits (serving/day)	1.71 (1.15)	1.90 (1.61)	**0.038**
Fruit juices (serving/day)	0.04 (0.11)	0.04 (0.17)	0.719
High-energy beverages (serving/day)	0.30 (0.47)	0.20 (0.28)	**0.003**
French fries (serving/day)	0.08 (0.19)	0.10 (0.16)	0.825
Coffee (serving/day)	0.00 (0.01)	0.00 (0.03)	0.331
Snacks (serving/day)	0.57 (0.87)	0.60 (0.73)	0.910

IQR: interquartile range

Using Mann-Whitney test

ORs and 95% CIs in the crude and adjusted models across the tertiles of EDIH and ELIH are shown in Table 3. As can be observed, the odds of CRC in the last tertile of ELIH increased significantly compared to the first tertile in the crude model (OR = 2.44; 95% CI: 1.18–5.05). In the adjusted model, the odds of CRC in the last tertile compared to the first tertile showed a significant increase in both EDIH and ELIH (OR = 3.12; 95% CI: 1.30–7.48 and OR = 4.72; 95% CI: 1.15–19.39, respectively).

**Table 3:** Association between the EDIH and ELIH with colorectal cancer

Tertiles of Indices	Case/Control	Model 1			Model 2	
		OR	95% CI		OR	95% CI
**EDIH**						
T_1_ (≤ 0.34)	18/53	1.00	Ref.		1.00	Ref.
T_2_ (0.35–0.52)	25/46	1.60	0.77–3.29		2.16	0.93–4.98
T_3_ (≥ 0.53)	28/43	1.91	0.93–3.92		**3.12**	**1.30–7.48**
P_trend_		0.076			**0.018**	
**ELIH**						
T_1_ (≤ 1.16)	16/53	1.00		Ref.	1.00	Ref.
T_2_ (1.17–1.33)	24/47	1.69		0.80–3.56	2.26	0.81–6.31
T_3_ (≥ 1.34)	31/42	**2.44**		**1.18–5.05**	**4.72**	**1.15–19.39**
P_trend_		**0.016**			**0.024**	

EDIH: empirical dietary index for hyperinsulinemia, ELIH: empirical lifestyle index for hyperinsulinemia, OR: odds ratio, CI: confidence interval, T: tertile, Ref: reference

Obtained from logistic regression

These values are odds ratio (95% CIs).

Significant values are shown in bold

Model 1: crude model

Model 2: adjusted for smoking, BMI, physical activity, energy intake, family history of CRC in first- and second-degree relatives, and taking ibuprofen and aspirin

## Discussion

Our findings demonstrated a significant positive relationship between EDIH and ELIH scores and CRC odds after adjusting for some potential confounders (smoking, BMI, physical activity, energy intake, family history of CRC in first- and second-degree relatives, and taking ibuprofen and aspirin.

The association between diet, insulinemic potential, and CRC risk was previously evaluated [23, 24]. II is one of the first indices whose relationship with CRC risk and CRC survival has been investigated in previous studies [23, 24]. However, II evaluates the diet’s short-term (postprandial) effect on insulin response. The other indices that measure the insulinemic potential of diet and lifestyle are EDIH and ELIH scores. EDIH predicts hyperinsulinemia using C-peptide concentration that involves food groups associated with insulin biomarker responses based on dietary intake over the long term. As previously mentioned, in addition to food groups associated with insulin biomarkers, BMI and physical activity are components of the ELIH [12].

The association between these lifestyle and dietary indices and the risk of different types of cancer, including CRC, was previously assessed [25–27]. A recent study conducted using the HPFS and the NHS data demonstrated that CRC risk was 33% higher in men in the higher quintile of EDIH compared to the lower. Also, the risk of CRC was 22% higher for women and 26% higher for the total population when the highest quantile of EDIH was compared to the lowest quantile [13]. Since the effect of gender on the odds of developing CRC was not investigated in the present study, it is not possible to compare the mentioned study with the present study. The NHSII cohort study demonstrated that the risk of CRC increased by 67% and 51% when the highest quantile of EDIH and ELIH was compared with the lowest quantile, respectively. Additionally, this study discovered a more powerful association between ELIH and the early onset of CRC and EDIH and CRC risk after age 50 [14]. That may be due to obesity being considered in the ELIH score but not in the EDIH score. According to research conducted among women, obesity was related to an increased risk of early onset of CRC [28]. The other study that assessed the association between a high insulinemic diet and CRC survival found a 66% higher risk of death from CRC in patients with the highest EDIH score than in the lowest quantile. Also, patients who continued to eat insulinemic diets were 51% more likely to die from CRC before and after diagnosis [15]. As a result, our results align with previous studies’ findings and show the effect of the two studied indicators on increasing the odds of CRC.

The role of hyperinsulinemia in CRC development was previously evaluated. The bioavailability and expression of insulin-like growth factor-1 (IGF-1) are enhanced by hyperinsulinemia, consequently increasing cell proliferation and reducing apoptosis [29, 30]. In addition, colorectal epithelial cells receive signals from mitogenic and pro-angiogenic insulin, possibly making their metabolism more active [31]. The association between hyperinsulinemia and increased C-peptide level, which is a marker for long-term secretion of insulin and insulin resistance, with CRC risk was reported in many previous studies [32–34]. So, exogenous hyperinsulinemia induced by the diet (with insulinemic potential) increases the risk of CRC. A validation study for EDIH score revealed that the subject’s C-peptide level varied across EDIH quintiles stratified by combined BMI and physical activity categories [12]. Overweight or obese and sedentary subjects had the highest C-peptide level across quantiles of EDIH score, while lean and active subjects had the lowest C-peptide concentration [12]. Circulating insulin levels have been linked to adiposity and physical activity levels [35, 36], which are involved in the development of CRC, and being overweight or less active is linked to a higher risk of CRC [37–39]. Reduced circulating levels of insulin and bioavailability of IGF-I are linked to higher physical activity levels; they are mitogenic hormones that are associated with cancer formation [36].

This study had strengths. The current study was the first case-control study to assess the association between dietary and lifestyle indices with insulinemic potential and the odds of CRC in the Middle Eastern population. Dietary intake of the Middle-Eastern population has its own unique pattern: high consumption of refined grains with large portion sizes and a higher percentage of energy from carbohydrates [40]. In addition, we used the food-based EDIH and ELIH scores, which correlates with circulating C-peptide levels. Also, we collected data for some important covariates, including non-steroidal anti-inflammatory drug (NSAID) consumption. Moreover, we matched subjects of case and control groups regarding sex and age, decreasing the potential of residual confounding factors. However, the limitations of this study should be considered. In self-reported data, measurement error regarding diet and lifestyle was possible. Also, some unmeasured variables (residual confounders) play a role as confounding factors and will likely affect our findings.

## Conclusions

The results of the present study revealed that people who were in the highest tertile of EDIH and ELIH had a higher odds of developing CRC compared to people who were in the lowest tertile. As a result, the chance of CRC increases with high EDIH and ELIH. Therefore, lifestyle and diet with insulinemic potential can influence the CRC odds, and dietary intervention to recommend a low insulinemic potential diet may prevent CRC risk.

### Electronic supplementary material

Below is the link to the electronic supplementary material.

Supplementary Material 1

Supplementary Material 2

Supplementary Material 3

## Data Availability

The datasets used and/or analyzed during the current study are available from the corresponding author on reasonable request.
