# Supplementary material for: Dietary and lifestyle indices for hyperinsulinemia and colorectal cancer risk: a case-control study
Source: BMC Gastroenterol. 2023 Dec 11;23:434. doi: 10.1186/s12876-023-03073-y (PMC10712032; doi:10.1186/s12876-023-03073-y)
Supplement: Supplementary file 1 — Supplementary Material 1 [file 12876_2023_3073_MOESM1_ESM.docx]

**Supplementary 1**

In the Name of God

Research Institute of Endocrinology and Metabolism

Shaheed Beheshti University of Medical Sciences

Food Frequency Questionnaire (FFQ)

Name: Acceptance Code: Age:

| No. | DAILY FOODS | HOW MUCH |  | HOW | OFTEN |  | CONSIDERATIONS |
| --- | --- | --- | --- | --- | --- | --- | --- |
|  |  |  | per day | per week | per month | per year |  |
| 1 | Lavash bread (refined grains) | 1 loaf |  |  |  |  |  |
| 2 | Barbari bread (refined grains) | 1 loaf |  |  |  |  |  |
| 3 | Sangak bread (whole grains) | 1 loaf |  |  |  |  |  |
| 4 | Taftoon bread (refined grains) | 1 loaf |  |  |  |  |  |
| 5 | Baguette bread | 1 loaf |  |  |  |  |  |
| 6 | Toast Bread | 1 slice |  |  |  |  |  |
| 7 | Cooked rice | 1 dinner plate |  |  |  |  | normal: full: |
| 8 | Cooked pasta | 1 spatula |  |  |  |  | Flat head: full: |
| 9 | Potato | 1 medium |  |  |  |  |  |
| 10 | French fries | 1 slice |  |  |  |  |  |
| 11 | Baked vermicelli (soup noodle) | 1 cup |  |  |  |  |  |
| 12 | Ash noodle | 1 cup |  |  |  |  |  |
| 13 | Wheat Flour |  |  |  |  |  |  |
| 14 | Cookies (stating the type) | 1 number |  |  |  |  | type: |
| 15 | Crackers (crispy biscuits) | 1 number |  |  |  |  |  |
| 16 | Cake Yazdi | 1 medium slice |  |  |  |  |  |
| 17 | Homemade Cake | 1 medium slice |  |  |  |  |  |
| 18 | Simple Cake | 1 medium slice |  |  |  |  |  |
| 19 | Corn | 1 medium |  |  |  |  |  |
| 20 | Cooked barley or bulgur | 1 cup |  |  |  |  |  |
| 21 | Lentil | 1 cup |  |  |  |  |  |
| 22 | Beans | 1 cup |  |  |  |  |  |
| 23 | Pea | 1 cup |  |  |  |  |  |
| 24 | Baked bean | 1 cup |  |  |  |  |  |
| 25 | Soy bean | 1 cup |  |  |  |  |  |
| 26 | Mung | 1 cup |  |  |  |  |  |
| 27 | Cotyledon | 1 cup |  |  |  |  |  |
| 28 | Beef or calf | 1 slice of stewed |  |  |  |  |  |
| 29 | lamb meat | 1 slice of stewed |  |  |  |  |  |
| 30 | Ground beef | 1 tablespoon |  |  |  |  |  |
| 31 | Chicken with skin | 1 medium piece |  |  |  |  |  |
| 32 | Skinless chicken | 1 medium piece |  |  |  |  |  |
| 33 | Fish (except tuna) stating the type | 1 medium piece (one palm full) |  |  |  |  | The amount of: type: |
| 34 | Tuna (canned) | 1/2 cans |  |  |  |  | Is it discarded oil? Yes * No * |
| 35 | Heart, liver and kidney | 1 skewer |  |  |  |  |  |
| 36 | Hamburger | 1 number |  |  |  |  |  |
| 37 | Kielbasa | 1 cut |  |  |  |  |  |
| 38 | Sausage | 1 number (Germany * cocktails *) |  |  |  |  |  |
| 39 | Egg | 1 number |  |  |  |  |  |
| 40 | Tripe and Rennet | 1 piece |  |  |  |  |  |
| 41 | Tongue | 1 whole number |  |  |  |  |  |
| 42 | Brain | 1 whole number |  |  |  |  |  |
| 43 | Head | 1 palm |  |  |  |  |  |
| 44 | Leg | 1 number |  |  |  |  | If certain amount and components is used, noted. |
| 45 | Pizza | 1 number |  |  |  |  |  |
| 46 | Milk skimmed | 1 cup |  |  |  |  |  |
| 47 | Low-fat milk (less than 2%) | 1 cup |  |  |  |  |  |
| 48 | Whole milk (greater or equal to 2%) | 1 cup |  |  |  |  |  |
| 49 | Cocoa milk | 1 cup |  |  |  |  |  |
| 50 | Chocolate milk | 1 cup |  |  |  |  |  |
| 51 | Strained yogurt | 1 tablespoon |  |  |  |  |  |
| 52 | Normal yogurt | 1 bowl/cup |  |  |  |  |  |
| 53 | Full-fat yogurt | 1 bowl/cup |  |  |  |  |  |
| 54 | Creamy yogurt | Half cup |  |  |  |  |  |
| 55 | Cheese | 1 pat |  |  |  |  |  |
| 56 | Cream cheese | 1 pat |  |  |  |  |  |
| 57 | Dough | 1 cup |  |  |  |  |  |
| 58 | Cream | 1 tablespoon |  |  |  |  |  |
| 59 | Traditional ice cream | Half cup |  |  |  |  | How months of year? |
| 60 | Non-traditional ice cream | 1 number |  |  |  |  | How months of year? |
| 61 | Butter | 1 pat |  |  |  |  |  |
| 62 | Margarine | 1 pat |  |  |  |  |  |
| 63 | Dried whey | 1 tablespoon |  |  |  |  |  |
| 64 | Shredded lettuce | 1 cup |  |  |  |  |  |
| 65 | Tomato | 1 medium |  |  |  |  |  |
| 66 | Cucumber | 1 medium |  |  |  |  |  |
| 67 | Fresh herbs | 1 small plate |  |  |  |  |  |
| 68 | Cooked vegetables (soup, rice, etc.) | 1 cup |  |  |  |  |  |
| 69 | Pumpkin | 1 medium |  |  |  |  |  |
| 70 | Stewed pumpkin | 1 medium |  |  |  |  |  |
| 71 | Baked eggplant | 1 medium |  |  |  |  |  |
| 72 | Boiled celery | 1 cup |  |  |  |  |  |
| 73 | Cooked green peas | 1 cup |  |  |  |  |  |
| 74 | Cooked green beans | 1 cup |  |  |  |  |  |
| 75 | Raw carrots | 1 medium |  |  |  |  |  |
| 76 | Cooked carrots | 1 medium |  |  |  |  |  |
| 77 | Garlic | 1 clove |  |  |  |  |  |
| 78 | Raw onion | 1 small |  |  |  |  |  |
| 79 | Fried onions | 1 tablespoon |  |  |  |  |  |
| 80 | Cabbage varieties | 1 bowl/cup |  |  |  |  |  |
| 81 | Bell peppers | 1 medium |  |  |  |  |  |
| 82 | Cooked spinach | 1 cup |  |  |  |  |  |
| 83 | Raw spinach | 1 cup |  |  |  |  |  |
| 84 | Rob | 1 tablespoon |  |  |  |  |  |
| 85 | Turnip | 1 medium |  |  |  |  |  |
| 86 | Small green pepper | 1 medium |  |  |  |  |  |
| 87 | Ketchup | 1 tablespoon |  |  |  |  |  |
| 88 | Pickles | 1 cup |  |  |  |  | How months of year? |
| 89 | SHOOR | 1 cup |  |  |  |  | How months of year? |
| 90 | Pickled cucumber | 1 medium |  |  |  |  |  |
| 91 | Cantaloupe | 1/4 number |  |  |  |  |  |
| 92 | Melon | 1 medium slice |  |  |  |  |  |
| 93 | Watermelon | 1 medium slice |  |  |  |  |  |
| 94 | Pear | 1 medium |  |  |  |  |  |
| 95 | Apricot | 1 medium |  |  |  |  |  |
| 96 | Cherries | 1 small plate |  |  |  |  |  |
| 97 | Apple | 1 medium |  |  |  |  |  |
| 98 | Peach | 1 medium |  |  |  |  |  |
| 99 | Nectarines | 1 medium |  |  |  |  |  |
| 100 | Prunes | 1 medium |  |  |  |  |  |
| 101 | Fresh figs | 1 medium |  |  |  |  |  |
| 102 | Dried figs | 1 medium |  |  |  |  |  |
| 103 | Grape | 1 medium bunch |  |  |  |  |  |
| 104 | Kiwi | 1 medium |  |  |  |  |  |
| 105 | Grapefruit | 1 medium |  |  |  |  |  |
| 106 | Orange | 1 medium |  |  |  |  |  |
| 107 | Persimmon | 1 medium |  |  |  |  |  |
| 108 | Tangerine | 1 medium |  |  |  |  |  |
| 109 | Pomegranate | 1 medium |  |  |  |  |  |
| 110 | Date | 1 medium |  |  |  |  |  |
| 111 | Plums (yellow and red) | 1 medium |  |  |  |  |  |
| 112 | Strawberry | 1 number |  |  |  |  |  |
| 113 | Sour cherry |  |  |  |  |  |  |
| 114 | Banana | 1 medium |  |  |  |  |  |
| 115 | Sweet lemon | 1 medium |  |  |  |  |  |
| 116 | Lemon | 1 medium |  |  |  |  |  |
| 117 | Orange juice | 1 cup |  |  |  |  |  |
| 118 | Apple juice | 1 cup |  |  |  |  |  |
| 119 | Grapefruit juice | 1 cup |  |  |  |  |  |
| 120 | Honeydew melon juice | 1 cup |  |  |  |  |  |
| 121 | Cornelian cherry | Half cup |  |  |  |  |  |
| 122 | Fresh pineapple | 1 Slice |  |  |  |  |  |
| 123 | Canned pineapple | 1 Slice |  |  |  |  |  |
| 124 | Cantaloupe | 1 medium slice |  |  |  |  |  |
| 125 | Raisins | 1 tablespoon |  |  |  |  |  |
| 126 | Fresh berries | 1 small plate |  |  |  |  |  |
| 127 | Dried berries | 1 number |  |  |  |  |  |
| 128 | Dried peach | 1 number |  |  |  |  |  |
| 129 | Dried apricot | 1 number |  |  |  |  |  |
| 130 | Green olive | 1 number |  |  |  |  |  |
| 131 | Canned fruits | 1 can |  |  |  |  |  |
| 132 | Packaged fruit juices | 1 number |  |  |  |  |  |
| 133 | Hydrogenated vegetable oil | 1 tablespoon |  |  |  |  |  |
| 134 | Liquid oil | 1 tablespoon |  |  |  |  |  |
| 135 | Olive oil | 1 tablespoon |  |  |  |  |  |
| 136 | Animal oil | 1 tablespoon |  |  |  |  |  |
| 137 | Tallow tail fat | 1 tablespoon |  |  |  |  |  |
| 138 | Mayonnaise | 1 tablespoon |  |  |  |  |  |
| 139 | Peanut | 1 number |  |  |  |  |  |
| 140 | Almond | 1 number |  |  |  |  |  |
| 141 | Walnut | 1whole number |  |  |  |  |  |
| 142 | Pistachios | 1 number |  |  |  |  |  |
| 143 | Hazelnut | 1 number |  |  |  |  |  |
| 144 | Seeds (watermelon, pumpkin, sunflower) | 1 bowl/cup |  |  |  |  |  |
| 145 | Sugar cube, comfit | 1 number |  |  |  |  |  |
| 146 | Sugar | 1 teaspoonful |  |  |  |  |  |
| 147 | Honey | 1 teaspoonful |  |  |  |  |  |
| 148 | Jams (by type) | 1 tablespoon |  |  |  |  |  |
| 149 | Industrial or cola beverages. | 1 cup |  |  |  |  |  |
| 150 | Dried sweets | 1 medium |  |  |  |  |  |
| 151 | Cream sweets | 1 medium |  |  |  |  |  |
| 152 | GAZ | 1 medium |  |  |  |  |  |
| 153 | SOHAN | 1 piece |  |  |  |  |  |
| 154 | Puff | 1 pack |  |  |  |  |  |
| 155 | Chocolate | 1 number |  |  |  |  |  |
| 156 | Crème caramel | 1 number |  |  |  |  |  |
| 157 | Tea | 1 cup |  |  |  |  |  |
| 158 | Salt | 1 tsp |  |  |  |  | Type: |
| 159 | Abgoosht | 1 cup |  |  |  |  |  |
| 160 | Chips | 1 pack |  |  |  |  |  |
| 161 | Coffee | 1 cup |  |  |  |  |  |
| 162 | Lemon juice | 1 teaspoonful |  |  |  |  |  |
| 163 | Candy | 1 medium |  |  |  |  |  |
| 164 | Baked mushrooms | Half cup |  |  |  |  |  |
| 165 | Homemade HALVA | 1 tablespoon |  |  |  |  |  |
| 166 | Sesame pudding | 1 tablespoon |  |  |  |  |  |
| 167 | Noghl | 10 number |  |  |  |  |  |
| 168 | Donuts | 1 number |  |  |  |  |  |

The date of delivery of food questionnaires to residents:

Name of dietitian responsible for completing FFQ:

Delivery date to Nutrition expert
