# Supplementary material for: Dietary and lifestyle indices for hyperinsulinemia and colorectal cancer risk: a case-control study
Source: BMC Gastroenterol. 2023 Dec 11;23:434. doi: 10.1186/s12876-023-03073-y (PMC10712032; doi:10.1186/s12876-023-03073-y)
Supplement: Supplementary file 2 — Supplementary Material 2 [file 12876_2023_3073_MOESM2_ESM.docx]

**Supplementary 2**

Components and weight of each of the EDIH and ELIH indices.

| **Empirical dietary index for hyperinsulinemia (EDIH)** | | **Empirical lifestyle index for hyperinsulinemia (ELIH)** | |
| --- | --- | --- | --- |
| Food Items | Weight | Food Items | Weight |
| **Positive association** | | **Positive association** | |
| French fries | 0.581 | Red meats | 0.089 |
| Red meats | 0.250 | Butter | 0.058 |
| Processed meats | 0.199 | BMI | 0.051 |
| Poultry | 0.183 | Fruit juices | 0.042 |
| Other fishes | 0.172 | Margarine | 0.041 |
| Eggs | 0.124 | **Reverse association** | |
| High-energy beverages | 0.104 | Salad dressing | -0.059 |
| Tomatoes | 0.095 | High-fat dairy products | -0.054 |
| Butter | 0.094 | Whole fruits | -0.029 |
| Margarine | 0.054 | Snacks | -0.024 |
| Low-fat dairy products | 0.025 | Coffee | -0.020 |
| **Reverse association** | | Physical activity | -0.001 |
| Green leafy vegetables | -0.055 |  | |
| High-fat dairy products | -0.046 |  |  |
| Coffee | -0.035 |  |  |
| Fruit juices | -0.029 |  |  |

BMI: body mass index.

^*^Weights are regression coefficients derived from the previous study by Tabung et al.
