# Supplementary material for: Dietary and lifestyle indices for hyperinsulinemia and colorectal cancer risk: a case-control study
Source: BMC Gastroenterol. 2023 Dec 11;23:434. doi: 10.1186/s12876-023-03073-y (PMC10712032; doi:10.1186/s12876-023-03073-y)
Supplement: Supplementary file 3 — Supplementary Material 3 [file 12876_2023_3073_MOESM3_ESM.docx]

**Supplementary 3**

In the Name of God

Research Institute of Endocrinology and Metabolism

Shaheed Beheshti University of Medical Sciences

| **Checklist** | | |
| --- | --- | --- |
| Code: | Hospital name: | Unit: |
| Group: Case / Control | Gender: Male / Female |  |
| First name: | Last Name: | Household income: |
| Age: | Age in Diagnosis: |  |
| Education level:  -No formal education  -Elementary  -Junior/Senior high school  -Diploma/College/University | Smoking:  -Never  -Former  -Current | Jobs:  -Employed/Government  -Employed/Private  -Housewife  -Retired  -Other |
| Family history of CRC in the first degree:  Yes  No | Family history of CRC in the second degree:  Yes  No | Mineral Supplement:  Yes  No |
| Ibuprofen:  Yes  No | Aspirin:  Yes  No | Acetaminophen:  Yes  No |
| Height: | Weight: | BMI: |
| Waist circumference: | Hip circumference: | Waist to hip ratio: |
| Common ways of cooking meat:  Raw / Fresh  Boiled  Fried, Fried / Frozen | Common ways of cooking vegetables:  Raw / Fresh  Boiled  Fried, Fried / Frozen |  |
